# To Choose or Not To Choose: Evaluating the Effect of a Choosing Wisely Knowledge Translation Initiative for Imaging in Low Back Pain by Emergency Physicians

**DOI:** 10.7759/cureus.4002

**Published:** 2019-02-04

**Authors:** Kavish Chandra, Paul R Atkinson, Hanif Chatur, Jacqueline Fraser, Cherie Lee Adams

**Affiliations:** 1 Emergency Medicine, Dalhousie University, Saint John, CAN; 2 Emergency Medicine, Saint John Regional Hospital, Saint John, CAN; 3 Emergency Medicine, Upper River Valley Hospital, Grafyon, CAN

**Keywords:** choosing wisely, low back pain, emergency medicine, knowledge translation, imaging

## Abstract

Introduction: We aimed to quantify the baseline familiarity of emergency medicine (EM) physicians with the Choosing Wisely Canada (CWC)-EM recommendations. We then assessed whether a structured knowledge translation (KT) initiative affected awareness, knowledge, and practice patterns for imaging in low back pain.

Methods: We completed a two-center, before and after practice evaluation study. Physicians working in two Canadian emergency departments (EDs) were asked to participate in a survey before a KT initiative, and were surveyed again at a six-month follow up period post-intervention. The primary outcome of physician practice was determined by analyzing the frequency of lumbar X-ray imaging for back pain.

Results: A total of 37 physicians were asked to complete the pre- and post-intervention survey. Awareness of the CWC-EM recommendations increased following the intervention (63%; 95%CI: 43-79 at baseline vs. 86%; 66-96 post-intervention). Knowledge increased with 58% (39-76) of physicians responding correctly initially, and 86% (66-96) after the intervention. Despite increases in awareness and knowledge of the guidelines, the lumbar X-ray imaging rate increased from a baseline of 12% (9.9-14.5) to 16.2% (13.6-19.2; p = 0.023) following the intervention.

Conclusion: We demonstrated some improvements in physician awareness and knowledge of the CWC-EM recommendations following our intervention. Despite these improvements, our KT intervention was associated with an increased frequency of imaging for low back pain, contrary to our expectations.

## Introduction

In Canada, healthcare expenditure has been predicted to reach $219 billion and comprises 10.9% of our gross domestic product [1]. Physicians play a role in rising healthcare costs with limited knowledge of the price of medical tests [2]. In order to provide effective patient care, physicians must have a working knowledge of recommended tests and therapies, as well as the frequency of harms and benefits of those interventions [3]. To that effect, physicians frequently overestimate the benefits and harms of medical interventions [3]. With similar trends seen in the USA, the Choosing Wisely (CW) campaign was launched by the American Board of Internal Medicine in 2012 [4-5]. Motivated by a demonstrated need, medical specialty societies in the USA were asked to identify tests and treatments that were overused and provided little benefit to patients. Modeled after the U.S. Choosing Wisely campaign, Choosing Wisely Canada (CWC) became a national campaign aimed to help patients and physicians critically think about medical tests, treatments, and procedures [6]. Since the inception of this now global campaign, studies are emerging characterizing the effectiveness of these recommendations. Rosenberg et al. [7] when reviewing multiple recommendations from multiple medical societies demonstrated a meager improvement, but largely no change in the frequency of medical imaging after the introduction of the CW recommendations. The data suggest that while the CW campaign is an innovative approach to help physicians and patients make effective choices to ensure high-quality care, a knowledge translation (KT) gap exists.

Knowledge translation is defined as the process of synthesizing scientific and practice information to create new understandings to aid clinicians treating patients [8]. It provides a means by which clinicians can organize, store, and access explicit knowledge [9]. It has become evident that publishing high-quality evidence alone may not change practice; as Wilson et al. [10] demonstrated that it can take up to 15 years from the time evidence is published to when it is routinely used in clinical practice. However, there is some data that practice and educational interventions may be helpful in closing that gap. In the emergency department (ED), the use of a computerized clinical decision support tool highlighting imaging indications consistent with the CW recommendations significantly decreased the number of computed tomography (CT) head imaging studies for mild traumatic brain injuries from 58.1% to 50.3% [11]. Amongst primary care physicians, one study reported 93.2% adherence to CW recommendations and this number increased to 96.5% following a one-hour educational seminar [12].

In Canada, rates of ED attendance are ranked among the highest globally with 17 million ED visits per year [13]. In 2015, the CWC campaign, along with the Canadian Association of Emergency Physicians (CAEP), developed the CWC-emergency medicine (EM) top five recommendations [14]. Since the first introduction of the original recommendations, five more recommendations have been added by CAEP [14]. Among the most common chief complaints is low back pain for adult patients, and there is considerable variability in physician management and treatment of this and other presentations [13]. This makes the practice of emergency physicians, especially with respect to the management of low back pain and imaging rates, an ideal topic to investigate the impact of a comprehensive KT intervention such as the CWC campaign.

There is a lack of data defining the awareness of physicians of the CWC-EM recommendations and its effect on physician practice. Therefore, we sought to quantify the baseline familiarity of the CWC-EM recommendations among physicians working in two EDs in New Brunswick using a survey. Our intervention was a structured KT initiative and our comparison were physicians in the same ED six months after our KT initiative. We wished to look at the awareness and knowledge of the CWC-EM recommendations, as well the primary outcome of frequency of lumbar X-ray imaging conducted for back pain, as a proxy for physician practice.

## Materials and methods

Assessing baseline awareness and knowledge

The ED physicians working in a community teaching hospital (Saint John Regional Hospital; SJRH) and rural teaching hospital (Upper River Valley Hospital; UVRH) were asked to participate in a survey assessing awareness and knowledge of the CWC-EM recommendations [14] (Table 1; March 2016, Appendix A). Content knowledge was classified as an ability of the respondents to identify 80% or more of the recommendations correctly. The pre-intervention survey was conducted before the KT intervention (April 2016).

**Table 1:** Choosing Wisely Canadian-Emergency Medicine (CWC-EM) recommendations. Recommendations 1-5 represent the original five recommendations released in June 2015 while 6-10 were released in October 2016.

	Ten things physicians and patients should question
1	Don’t order computed tomography (CT) head scans in adults and children who have suffered minor head injuries (unless positive for a validated head injury clinical decision rule)
2	Don’t prescribe antibiotics in adults with bronchitis/asthma and children with bronchiolitis
3	Don’t order lumbosacral (low back) spinal imaging in patients with non-traumatic low back pain who have no red flags/pathologic indicators
4	Don’t order neck radiographs in patients who have a negative examination using the Canadian C-spine rules
5	Don’t prescribe antibiotics after incision and drainage of uncomplicated skin abscesses unless extensive cellulitis exists
6	Don’t order CT head scans in adult patients with simple syncope in the absence of high-risk predictors
7	Don’t order CT pulmonary angiograms or VQ scans in patients with suspected pulmonary embolism until risk stratification with decision rule has been applied and when indicated, D-dimer biomarker results are obtained
8	Don’t use antibiotics in adults and children with uncomplicated sore throats
9	Don’t order ankle and/or foot X-rays in patients who have a negative examination using the Ottawa ankle rules
10	Don’t use antibiotics in adults and children with uncomplicated acute otitis media

Knowledge translation initiative

The KT intervention consisted of a structured package that included a didactic seminar reviewing the CWC-EM recommendations which was presented in person, and also electronically to all participating physicians, and made available on the departmental website [15]. In addition, CWC-developed posters reiterating the EM recommendations and aimed at patient and physician groups respectively were placed throughout the department in high-volume areas.

Assessing initial knowledge transfer

Physicians were subsequently surveyed six months following the implementation of the KT intervention in September 2016 (Poster: Chandra K, Fraser J, Chatur H, Atkinson P, Adams CL. To Choose or Not to Choose. CAEP Annual Conference; June 2017).

Assessing practice change

The effect of the intervention was determined by analyzing the frequency of imaging studies conducted for back pain before and after the introduction of our intervention. All patients presenting to the community ED with the primary Canadian Emergency Department Information System (CEDIS) complaint of back pain were included, and the lumbosacral X-ray tests from June to September 2014 for the pre-intervention period and June to September 2016 for the post-intervention period were collected and analyzed (Presentation: Chandra K, Fraser J, Chatur H, Atkinson P, Adams CL. The Contrarian Effect. CAEP Annual Conference; June 2017).

Data analysis

Based on previous literature, which showed an average X-ray imaging rate of 45% for low back pain, [7, 16] we calculated that 395 patients would be required in each group (pre- and post-intervention) to detect a 10% difference in imaging with a power of 0.80 and an alpha of 0.05. Following the end of the study period, data were analyzed using the Fisher’s exact tests and 95% CI were also calculated for both the survey and imaging data using GraphPad QuickCalcs (La Jolla, CA, USA). This study was approved by the Horizon Health Network Research Ethics Board in Saint John, New Brunswick, Canada (file number 2016-2275).

## Results

Assessing awareness and knowledge

A total of 36 physicians were invited to participate in the pre-intervention survey, and 37 in the post-intervention survey. The pre-intervention survey response rate was 67% and the post-intervention response rate was 59%.

**Figure 1:**
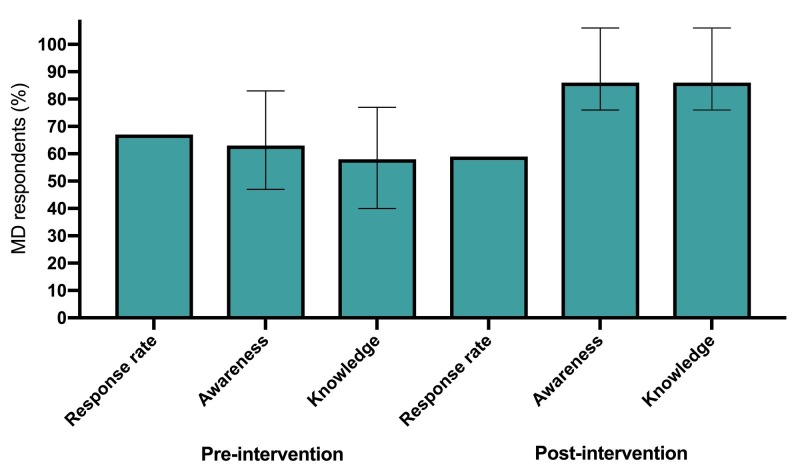
Physician awareness and knowledge of the Choosing Wisely Canada-Emergency Medicine (CWC-EM) recommendations.

Awareness of the CWC-EM recommendations at baseline was high (63%; 95% CI: 43-79), and increased following the intervention (86%; CI: 66-96; Figure 1). Knowledge increased with 58% (CI: 39-76) of physicians responding correctly initially, and 86% (CI: 66-96) after the intervention (Figure 1).

Assessing practice change

**Figure 2:**
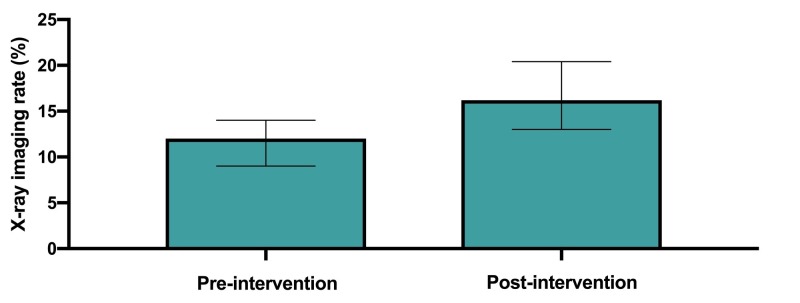
Frequency of lumbosacral X-rays for back pain.

Baseline characteristics of the included 1453 back pain patients were similar in the pre- and post-intervention groups (Table 2). At our SJRH site, there was a total of 781 visits for back pain from June to September 2014 and 672 from June to September 2016. For our primary outcome, the rate of lumbosacral X-ray imaging for back pain increased from a baseline of 94/781 (12%; CI: 10-15) before the intervention to 108/672 (16.2%; CI: 14-19; p = 0.023) following the intervention (Figure 2).

**Table 2:** Patient demographics in the pre- and post-intervention study groups for all included presentations with low back pain.

	Pre-intervention 2015	Post-intervention 2016
Total [emergency department (ED) visits for back pain]	781	672
Total number lumbar X-rays performed (n;%; 95% CI)	94 (12%; 9.9-14.5)	108 (16.2%; 13.6-19.2)
Age (mean+/-SD)	48 (27-68)	49 (29-70)
Male (%)	45	52
Monthly number of lumbar X-rays per number ED visits for back pain		
June	29/189	30/134
July	28/183	26/177
August	23/212	22/174
September	14/197	31/187

## Discussion

The first goal of our study was to establish the baseline awareness and knowledge of the CWC-EM recommendations, then reassess after a targeted KT initiative. As we hoped, we saw evidence of improved awareness and knowledge of the CWC-EM recommendations following our intervention. Our results are similar to those reported by Kost et al. [12] who demonstrated that CW adherence among primary care providers improved only after a one-hour seminar. Second, and somewhat surprisingly, we observed that our intervention was associated with a subsequent increase in the rate of imaging for low back pain. It is important to note that the baseline X-ray imaging rates for back pain in our study were lower than those that have been previously reported [7, 16]. Wilson et al. showed that the decision to order a particular test or treatment is based on a number of factors [10]. These factors can be the integration of the best available evidence, care provider experience, and patient values. Furthermore, the practice of not over-investigating back pain without red flags has been fairly well established among generalists. As evidenced by our results, the strong baseline rate of adherence to the CWC-EM recommendation likely reflects an already established commitment to high-quality medical care among emergency physicians in our study.

We were surprised to see the small but significant increase in the frequency of lumbar X-rays, following the KT intervention from 12% to 16.2%. One possible explanation for this unexpected increased rate of imaging could be due to a contrarian effect. This is the notion that when individuals are presented with a direct order not to do something, which is deliberate in the CWC terminology, they may potentially do the opposite, even if subconsciously. While not directly referred to in the literature, following the implementation of the Ottawa foot and ankle clinical decision rule, there was a small but significant increase in foot and ankle radiography in the control group [17]. Alternatively, the increase in lumbar X-rays could be due to an increased awareness of the recommended indications for ordering imaging for back pain, and the highlighting of the clinical red flags that would alert clinicians to other serious causes of back pain which may not have been factored into previous decisions to request imaging. We do not have data to determine if baseline imaging requests were low due to local practice patterns or because of high adherence to evidence-based guidelines. Reminders about red flags for back pain could inadvertently increase the imaging rate in all patients with low back pain, whether considered to be appropriate or inappropriate.

Although the principles underpinning the CWC are agreeable to most clinicians, it remains unclear if implementation of such guidance improves clinical practice. Green concluded that some clinical decision rules offer very little over solid professional judgment and if we adopt them, we need to scrutinize all the strengths and limitations [18]. Our study demonstrates that in a local clinical setting with low imaging rates and high adherence to established practice guidelines, implementation of national campaigns to further improve practice should be done cautiously employing an a priori needs assessment to avoid contrarily decreasing adherence.

Limitations

There are several limitations to our study. While assessing physician practice, our study assumed that all patients were triaged appropriately to the CEDIS complaint of back pain. It is possible some patients could have been more appropriately triaged to traumatic back/spinal injury if there was a history of trauma and, therefore, more appropriately imaged. Furthermore, our study assessed only the rate of lumbar X-rays, but not other advanced imaging modalities. Another potential limitation was that individual physician practices were not examined, and that the group effect of imaging was measured. While individual practices could confound results, there were no significant staff changes or other guideline or education changes other than the intervention itself.

## Conclusions

This study demonstrates that awareness and knowledge of the CWC-EM recommendations can be improved and sustained at six months by standard KT initiatives among physicians practicing in community and rural ED settings. Despite this increase in awareness and knowledge, we observed an increased frequency of X-ray requests for low back pain following our intervention. This calls into question the role of introducing national KT initiatives where rates of baseline local adherence to best practice guidelines are already high.

## Appendices

Appendix A: Physician survey

1.     Are you aware of the Choosing Wisely Canada-emergency medicine (CWC-EM) recommendations?

A: Yes   B: No

2.     Pick the five recommendations that are part of the CWC-EM recommendations.

A: Don’t order CT head scans in adults and children who have suffered minor head injuries unless positive for a head injury clinical decision rule

B: Don’t order a D-dimer in patients with a low probability of venous thromboembolism

C: Don’t prescribe antibiotics in adults with bronchitis/asthma and children with bronchiolitis

D: Don’t order lumbosacral (low back) spinal imaging in patients with nontraumatic low back pain who have no red flags/pathologic indicators.

E: Don’t routinely order an urinary culture in patients without lower urinary tract symptoms

F: Don’t routinely order post-reduction plain films

G: Don’t order neck radiographs in patients who have a negative examination using the Canadian C-spine rules

H: Don’t prescribe antibiotics after incision and drainage of uncomplicated skin abscesses unless extensive cellulitis exists

I: Don’t routinely give IV proton pump inhibitors to patients with abdominal pain without GI bleeding

J: Don’t routinely order INR/PTT in patients when getting other lab investigations

(bolded items are part of the CWC-EM recommendations)
